# Accuracy Analysis in Sensor Networks for Asynchronous Positioning Methods

**DOI:** 10.3390/s19133024

**Published:** 2019-07-09

**Authors:** Rubén Álvarez, Javier Díez-González, Efrén Alonso, Laura Fernández-Robles, Manuel Castejón-Limas, Hilde Perez

**Affiliations:** 1 Positioning Department, Drotium, Universidad de León, 24071 León, Spain; 2 Department of Mechanical, Computer and Aerospace Engineering, Universidad de León, 24071 León, Spain

**Keywords:** sensor networks, TDOA, asynchronous, Cramér–Rao lower bound, heteroscedasticity

## Abstract

The accuracy requirements for sensor network positioning have grown over the last few years due to the high precision demanded in activities related with vehicles and robots. Such systems involve a wide range of specifications which must be met through positioning devices based on time measurement. These systems have been traditionally designed with the synchronization of their sensors in order to compute the position estimation. However, this synchronization introduces an error in the time determination which can be avoided through the centralization of the measurements in a single clock in a coordinate sensor. This can be found in typical architectures such as Asynchronous Time Difference of Arrival (A-TDOA) and Difference-Time Difference of Arrival (D-TDOA) systems. In this paper, a study of the suitability of these new systems based on a Cramér-Rao Lower Bound (CRLB) evaluation was performed for the first time under different 3D real environments for multiple sensor locations. The analysis was carried out through a new heteroscedastic noise variance modelling with a distance-dependent Log-normal path loss propagation model. Results showed that A-TDOA provided less uncertainty in the root mean square error (RMSE) in the positioning, while D-TDOA reduced the standard deviation and increased stability all over the domain.

## 1. Introduction

Over the past few years, positioning systems have experienced a growing importance due to the wide range of applications they present in numerous civil and military fields. Positioning methods based on satellite systems, e.g., global navigation satellite systems (GNSS), offer accurate precision with global coverage but still present accuracy issues for specific crucial tasks such as high-precision trajectories or indoor navigation. These issues have recently attracted much attention with the advent of unmanned transportation.

Positioning systems have traditionally been classified into four main groups: Time of Arrival systems (TOA) [1,2], Time Difference of Arrival systems (TDOA) [3,4], Angle of Arrival systems (AOA) [5,6], Received Signal Strength Indication systems (RSSI) [7,8], or a combination of them [9,10]. Methods based on time measurement (i.e., TOA and TDOA) have been the main exponents of recent developments, on account of their robustness, universality, and reliability, in addition to their great accuracy and relative simplicity.

Time measurements have usually been obtained in two different ways. The most common one measures time intervals by synchronizing emitter and receiver clocks, which is mandatory in the case of TOA systems. The other option comes from the synchronization of receivers, and is optional in TDOA systems. This fact significantly affects the accuracy of the positioning determination process due to the appearance of clock instabilities and the introduction of small time-offsets during the process of synchronization among elements.

Owing to the challenging accuracy requirements to be met in sensor networks, the minimization of key factors that increase the uncertainty associated with the calculation of the position is an imperative task. Location systems based on TOA processes present disadvantages in this matter. Their accuracy may be in the order of 10 cm [11,12,13], but time errors which can occur during the synchronization process significantly increase the uncertainties associated with the position calculation [14,15,16].

Conventional TDOA methods have been traditionally dependent on global synchronization amongst the receivers, reaching higher accuracy levels requiring less energy than TOA systems. Nevertheless, synchronization instabilities are still present and the system complexity is higher than TOA architectures.

Notwithstanding these previous tendencies, a new pattern has been developed over the last few years, which advocates for the implementation of asynchronous architectures wherein a single clock is used to measure the time differences of arrival characteristic of TDOA systems. These new systems could overcome synchronization disadvantages in TDOA systems with less architecture complexity and higher sensor ubication flexibility, and they are key to this work. The main advantages of these new methods would include the elimination of the synchronization among receivers and the resulting error introduced in the measurement process [17,18].

In recent years, two different asynchronous systems have been proposed. Asynchronous TDOA [17] and difference-time TDOA [18]. These systems display an architecture based on a coordinator sensor and a collection of worker sensors. The coordinator sensor is in charge of processing the time measurements. The worker sensors collaborate in the determination of time differences. These new architectures avoid the necessity of a clock built into every sensor of the system. This fact reduces the overall costs and the complexity of the system and boosts accuracy by eliminating the interaction among sensor clocks. All these factors enhance indoor and low-level flight sensor positioning accuracy, a key factor that is driving the increasing popularity of these methods.

The aim of this article was to develop for the first time, to the extent of our knowledge, a methodology to select the suitability of these two asynchronous TDOA systems under different sensor placements in a 3D, real environment. This methodology must consider that vehicle navigation in local positioning systems (LPS) is highly affected by noisy environments, thus system evaluations must be performed at a high accuracy level even where time measurements are corrupted by noise.

Cramér–Rao Lower Bound (CRLB) is a commonly used estimator to determine the lowest possible uncertainty associated with a positioning process in line-of-sight (LOS) [19,20] and non-line-of-sight (NLOS) [21] conditions. This method models measurement uncertainties through the variance associated to every sensor range estimation.

Conventional models consider the presence of a constant variance associated to each time measurement [22]. However, to attain a higher level of accuracy in the results, it is imperative to introduce the distance between emitter and receiver in the model allowing for heteroscedasticity in time measurements [23,24]. This phenomenon is especially important in cases with medium and high signal-to-noise ratios (SNRs) in the receivers. If the SNR is reduced, the positioning pulse detection becomes significantly hampered, leading to drastic time measurement errors which reduce the accuracy of the positioning system [25].

Therefore, the hypothesis of heteroscedastic variances needs to be implemented on the basis of a propagation loss model over the signal path between the emitter and the receivers [26,27], depending on the positioning methodology and the characteristics of communication amongst every single sensor of the system.

The remainder of this paper is organized as follows. In Section 2, a comparison of the main characteristics of asynchronous TDOA architectures studied is developed. Section 3 covers the main path-loss models used to calculate the SNR. Section 4 includes a study of the Cramér–Rao Lower Bound, based on a matrix model implemented for each system architecture. Section 5 gathers the conclusions obtained from all the studies, along with the election of the best system. Lastly, Section 6 presents the conclusions and completes the article.

## 2. Asynchronous TDOA Methods

In this section, the asynchronous TDOA methods will be introduced. Neither of these methods need to synchronize any element of the system, using only one clock to measure the differences in times of the TDOA system. The notation used throughout the study is the one described hereafter: TS defines the location of the Target Sensor, Coordinate Sensor (CS) represents the position of the receiver in charge of the time measurements, Worker Sensors (WS) are the rest of emitters/receivers, *N* is the total number of WS, and additionally, a CS must be considered, $t_{START}$ and $t_{END}$ represent the start and the end of the time measurement process in the CS.

### 2.1. A-TDOA

**Figure 1:**
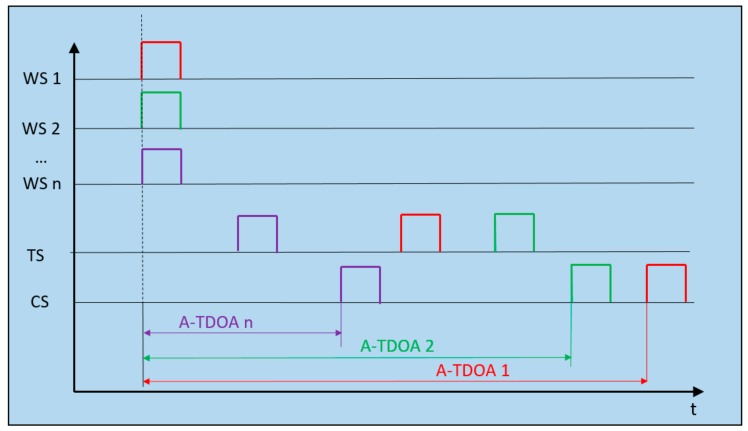
Asynchronous Time Difference of Arrival (A-TDOA) system timing diagram. Example of architecture operation with *n* Worker sensors (WS) nodes (*n* must be at least equal to 4). Rectangular positioning pulses are emitted from the WS nodes, and when the arrival of the signal to the Target Sensor (TS) node is produced, signals are instantaneously retransmitted to the Coordinate Sensor (CS) node. When the process is completed, A-TDOA time measurements are accomplished.

In Figure 1, an A-TDOA architecture is presented [17]. It proposes a passive positioning system based on one single clock in the CS node, using the positioned sensor as signal repeater.

Positioning pulses are emitted by WS nodes, reaching the CS node with successive time differences which lead to the beginning of the time measurement associated with each WS–CS ($t_{START_{i}}$). Conversely, the signal emitted by each WS node is received by the TS node in charge of sending again these signals to the Cs node ($t_{END_{i}}$). When the signal is received, the time measurement process comes to an end, completing the time measurement process of each WS–CS pair. The TDOA measurement in terms of distance is represented by the following relation.

(1) $$A-TDOA_{i}=c\left(t_{END_{i}\;}-\;t_{START_{i}}\right)-\|WS_{i}-CS\|\;i\in \left[1,N\right]$$

### 2.2. D-TDOA

**Figure 2:**
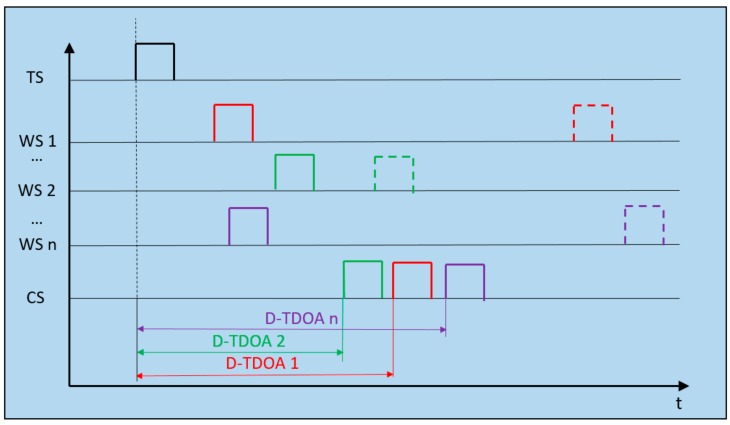
Difference-Time Difference of Arrival (D-TDOA) timing diagram. Example of architecture operation with *n* WS nodes (with a minimum number of 4). The positioning target pulse is received at every WS node of the system that retransmits it towards the CS node. D-TDOA time measurements are completed by a round-trip transmission (RTT) process between each pair of WS–CS nodes.

The D-TDOA method is based on the combination of a traditional TDOA system and a round trip time (RTT) method, accomplishing the goal of obtaining the time difference measurements with only one clock [18], as shown in Figure 2.

Positioning pulses are broadcast by the TS node, reaching the CS node at $t_{START}.$ This temporal reference is common to all the time measurements realized by the method. Meanwhile, the TS node emission is received at the WS nodes, which resend it to the CS node ($t_{END_{i}}$), completing the time difference measurement of each WS–CS pair of nodes. Lastly, the pulse is emitted from the CS node to WS nodes with the aim of calculating the processing time on each WS node. The TDOA measurement in terms of distance is represented by the following relation:

(2) $$D-TDOA_{i}=c\left(t_{END_{i}}-t_{START}\right)-\|WS_{i}-\;CS\|,i\in \left[1,N\right]$$

While $A-TDOA_{i}$ and $D-TDOA_{i}$ hold similar equations, the time measurement recordings and positioning pulse travels differ significantly from one method to the other. These characteristics are analyzed in the following sections.

## 3. Heteroscedastic Noise Model

In this section, a noise variance distance-dependent model is implemented associated to the process of location in asynchronous TDOAs system, allowing for a better reproducibility of real conditions.

The process starts with the TOA variance range estimate due to the noisy environments. Next, the TDOA variance range estimate is defined based on time measurement correlation assumptions. The heteroscedastic noise variance model is completed with the path-loss propagation model implementation that best fit with the multiple sensor network’s characteristics.

Amongst the main sources of ranging errors of positioning systems based on time measurements, the most important for TDOA asynchronous techniques is the uncertainty induced by white Gaussian noise (WGN) in the propagation channel. This problem has been deeply studied for TOA architectures, quantifying it with the CRLB [25]:

(3) $$\sigma_{i}{}^{2}\geq \frac{c^{2}}{{\left(2\pi B\right)}^{2}\cdot t_{s}B\cdot SNR_{i}}\;,\;i=1,\;\ldots ,M$$

where ***M*** is the total number of sensors, $\sigma_{i}{}^{2}$ is the receptor range estimate variance, *c* is the pulse propagation velocity, B is the bandwidth, *t_S_* is the length of time during which the bandwidth is occupied, and $SNR_{i}$ is the signal-to-noise ratio for each receiver.

The majority of range estimation architectures consider the product $t_{s}B$ approximately unitary. This, together with the hypothesis of high levels of SNR at the receivers and efficient estimator, enables the derivation of the following relation for a TOA variance estimation [23,24].

(4) $$\sigma_{i}{}^{2}=\frac{c^{2}}{B^{2}\cdot SNR_{i}}\;,\;i=1,\;\ldots ,M$$

Based on the TOA variance estimation, the implementation for TDOA systems is made under some assumptions. He and Dong [17] proposed that the time measurements in asynchronous TDOA architectures are considered independent. Consequently, the off-diagonal elements of the covariance matrix associated with the Gaussian noise modelling are null. According to Kaune et al. [19], the variance associated with a difference distance between two nodes is the sum of the variances for each node, under the assumption of uncorrelated time measurements. 

Hence:

(5) $$\begin{array}{c} \sigma_{ij}{}^{2}=\sigma_{i}{}^{2}+\sigma_{j}{}^{2}\\ i=1,\;\ldots ,\;M\\ j=1,\;\ldots ,M \end{array}$$

Consequently,

(6) $$\begin{array}{c} \sigma_{ij}{}^{2}=\frac{c^{2}}{B^{2}}\left(\frac{1}{SNR_{i}}+\frac{1}{SNR_{j}}\right)\\ \;i=1,\;\ldots ,\;M\\ j=1,\;\ldots ,\;M \end{array}$$

In Equation (6), the *SNR* associated with each receiver mainly depends on the power emission, the transmission frequency, and environment. This last aspect is characterized by means of path-loss propagation models for indoor and outdoor environments. Large-scale models predict the mean signal strength in LOS environments based on the distance between emitter/receiver and the characteristics of the signal. Small-scale or fading models characterize the rapid fluctuations of the signal when the distance of the emitter/receiver is short, in both conditions (LOS and NLOS) [27].

Multiple sensor networks with high location accuracy are used in many applications. However, in the majority of systems, path loss during operation presents a higher level of dependency on large distances of the emitter/receiver and LOS propagation.

Consequently, large-scale models seem more appropriate for this analysis. Assuming invariant power transmission and homogeneity in the operation of receivers, the following relations are established:

(7) SNRi= PRiPn=PTPLiPn=PTPn·1PLi i=1, …, N

where $P_{T}$ is transmission power, $P_{R_{i}}$ is the received power in each receiver, PL is the path loss, and $P_{n}$ is the mean noise power, obtained from the Johnson–Nyquist equation:

(8) $$P_{n}=\;k_{B}T_{0}B$$

where $k_{B}$ is the Boltzmann’s constant, $T_{0}$ is the absolute temperature of the receiver input, and B is the receiver bandwidth.

Large-scale path loss models have been deeply studied in the last decades for modelling mobile communications. The vast majority of these methods were built under some of these restrictions: emitter and receiver heights, transmission frequency, and emitter–receiver distance, among others. Under these limitations, the modelling of asynchronous TDOA architecture is not possible due to the emitter/receiver’s characteristics in multiple sensor networks.

Based on the preceding assumptions, the path loss propagation model selected for the simulation is the Log-normal, which eliminates the restrictions on emitter–receiver geometry.

(9) $$Log-normal:\;PL_{i}=\;PL\left(d_{0}\right)\;{\left(\frac{d_{i}}{d_{0}}\right)}^{n}$$

The noise model final implementation in the CRLB variance definition is presented below:

(10) σij2=c2B2PTPnPLd0did0n+djd0ni=1, …, Mj=1, …, M

where $d_{0}$ is the reference distance to the emitter, the basis from which the Log-normal model hypothesis is valid, $PL\left(d_{0}\right)$ is the path loss for this distance, and *n* is the path loss exponent.

## 4. CRLB Derivation for A-TDOA and D-TDOA Systems

The prediction of the uncertainty associated with the position calculation process is one of the most significant accomplishments in the design and development of positioning systems.

From a statistical point of view, CRLB expresses the minimum variance value of any unbiased estimator of a deterministic parameter. In other words, the CRLB defines the minimum possible uncertainty associated with an estimation process.

(11) $$var\left(\hat{\theta }\right)\geq \frac{1}{FIM}=\frac{1}{E\left[{\left[\frac{\partial }{\partial x}\ln f\left(X;\theta \right)\right]}^{2}\right]}$$

In this equation, $\hat{\theta }$ is the unbiased estimator for the parameter of study, *FIM* is the Fisher Information Matrix, *E* the expectation value of the denominator function, *X* the parameter measurement vector, θ is the parameter vector to be estimated, and $f\left(X;\theta \right)$ is the probability density function of *X* for the parameter θ.

Cramér-Rao Lower Bound has proved to be especially suitable in positioning. This is due to its definition based on a prior knowledge possibility of maximum reachable exactitude in terms of the architecture geometry, the environment modelling, and the intrinsic characteristics of measurement instruments. This maximum value reached by the position estimator would be valid as long as it is unbiased and efficient.

In this section, the CRLB is adapted to A-TDOA and D-TDOA architectures. Additionally, the noise variance model introduced in Section 3 is implemented in order to estimate the RMSE in the TS location estimation.

For a TDOA system, time measurements associated with the receivers are modelled by the addition of WGN. In a real environment, the variance associated with this phenomenon depends on the distance between emitter and receiver, inducing heteroscedasticity in data management. In this context, Kaune et al. [19] includes a model of the dependent parameter’s variance in the calculation of the inverse of the Fisher Information Matrix *(J*).

(12) $$J=\frac{1}{\sigma^{2}\left(TS\right)}{\left(\frac{\partial h\left(TS\right)}{\partial TS}\right)}^{T}\left(\frac{\partial h\left(TS\right)}{\partial TS}\right)+\frac{1}{2}\frac{1}{\sigma^{2}\left(TS\right)}{\left(\frac{\partial \sigma \left(TS\right)}{\partial TS}\right)}^{T}\left(\frac{\partial \sigma \left(TS\right)}{\partial TS}\right)$$

That can be expressed in matrix form as:

(13) $$J_{mn}={\left(\frac{\partial h\left(TS\right)}{\partial x_{m}}\right)}^{T}R^{-1}\left(TS\right)\left(\frac{\partial h\left(TS\right)}{\partial x_{n}}\right)+\frac{1}{2}tr\left(R^{-1}\left(TS\right)\left(\frac{\partial R\left(TS\right)}{\partial x_{m}}\right)R^{-1}\left(TS\right)\left(\frac{\partial R\left(TS\right)}{\partial x_{n}}\right)\right)$$

where sub-indexes ***m*** and ***n*** refer to the respective row and column of *J*. The column matrix *h(X)* expresses the differences in the Euclidean distances among the TDOA measurements of each pair of receivers:

(14) $$h_{A-TDOA}{}_{i}=\|TS-WS_{i}\|+\|TS-CS\|-\|WS_{i}-CS\|\;i=1,\;\ldots ,\;N$$

(15) $$h_{D-TDOA}{}_{i}=\|TS-WS_{i}\|+\|WS_{i}-CS\|-\|TS-CS\|\;i=1,\;\ldots ,\;N$$

Finally, *R(x)* is the covariance matrix of the system, which is characterized by null off-diagonal elements for both systems, due to the non-correlation among time measurements. The variance modelling was implemented according to the model explained in Section 3, with the following definition for the distances between each asynchronous TDOA system.

(16) $$\begin{array}{c} d_{A-TDOA_{\;i}}=\|TS-WS_{i}\|+\|TS-CS\|\\ d_{A-TDOA_{\;j=1}}=\;\|WS_{i}-CS\|\\ i=1,\;\ldots ,\;N \end{array}$$

(17) $$\begin{array}{c} d_{D-TDOA_{\;i}}=\|TS-WS_{i}\|+2\|WS_{i}-CS\|\\ d_{D-TDOA_{\;j=1}}=\;\|TS-CS\|\\ i=1,\;\ldots ,\;N \end{array}$$

The uncertainty is evaluated in terms of RMSE, as shown in the following equation (three-dimensional location), where the sub-indexes refer to the diagonal elements of matrix *J*:

(18) $$RMSE=\;\sqrt{J_{11}+J_{22}+J_{33}}$$

## 5. Simulation Results

In this section, asynchronous TDOA systems A-TDOA and D-TDOA are compared for sensor network positioning. Firstly, a set of global communication parameters are defined in Table 1.

In addition to these parameters, the comparison among architectures was carried out based on unity gain antennas in all system transceivers and on the assumption of full-duplex communication capacity among elements. Furthermore, an assumption of the receive-and-retransmit technique in transceivers operations and a unity frequency–time product in all the architectures’ communications was considered.

The results were obtained based on simulations carried out on an irregular surface of 1 km^2^ (1000 × 1000 m) with an elevation modelled by a normal distribution $N\left(15,9\right)$ cm. The space analysis was limited to a height above ground level from 1 to 100 m. Under this assumption, the spatial discretization was 100 m for surface coordinates (Cartesian *x* and *y*) and 10 m for coordinate *z*.

Additionally, the minimum height of sensor nodes (WS and CS) was restricted to 3 m with the objective of not inducing effects that were not considered by the Log-normal model (specially ground reflections and multipath). The maximum height was also limited to 13 m, but this restriction was related to the size of the supports (less than 10 m).

Lastly, a path loss exponent value of 2.1 was selected as highly recommended in sub-urban environments with medium frequencies [27]. Due to the theoretical approach of the problem, the Free Space Propagation Model (FSPM) was used to obtain $PL\left(d_{0}\right)$.

The comparison among systems (in Table 2) was carried out with five random distributions of receivers, each one with a number of five sensors. This was the minimum number of nodes for a unique three-dimensional location in TDOA architectures.

The best distributions for each system are illustrated in the following images, together with the CRLB evaluation in terms of RMSE at every point of the discretization.

**Figure 3:**
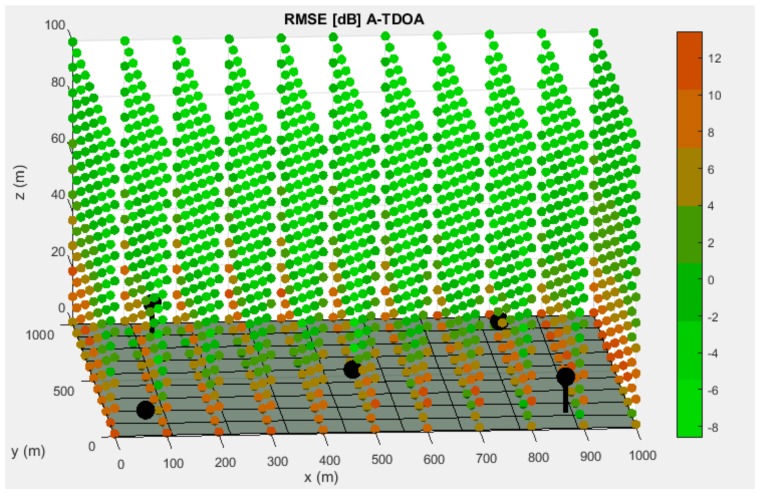
Best distribution of Worker Sensor (WS) and Coordinator Sensor (CS) nodes for the A-TDOA system. The base surface is presented as the grey hyperplane located at the bottom of the picture. The nodes are represented by black spheres with their correspondent holder that links them to the base surface. The CRLB evaluation of the discretization points is displayed according to the right-hand side legend.

**Figure 4:**
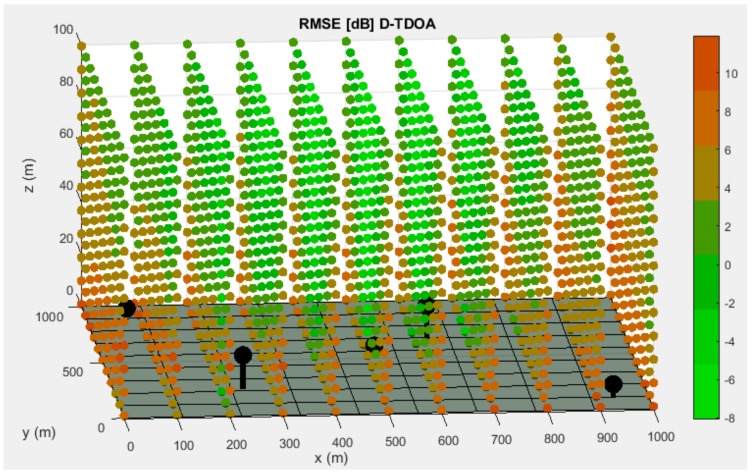
Best distribution of Worker Sensor (WS) and Coordinate Sensor (CS) nodes for the D-TDOA system. The base surface is presented as the grey hyperplane located at the bottom of the picture. The nodes are represented by black spheres with their correspondent holder that links them to the base surface. The CRLB evaluation of discretization points is displayed according to the right-hand side legend.

As it can be seen in Figure 3 and Figure 4, points that are close to the surface or nodes present a higher RMSE. This phenomenon is due to the relative location between nodes and these discretization points, that causes an increase of the influence of time measurements uncertainties in total positioning accuracy.

The final simulation results are presented in Table 3.

Based on the results of the simulations, it was shown that the A-TDOA system presented a lower mean RMSE value in every distribution. The minimum RMSE values in each distribution were obtained by the A-TDOA method. In the case of maximum RMSE values, the tendency was not obvious. Finally, it can be observed that the standard deviation in every distribution was lower for the D-TDOA system, which implies a higher stability.

In terms of architecture complexity, A-TDOA systems require an initial step in the emission of the positioning pulses of the WS nodes in order to simultaneously start the process. In contrast, in D-TDOA systems, the first communication link exclusively depends on the target node emission. Energy consumption is another factor to be evaluated. Due to the lower energy requirements for amplifying the signal power at the retransmission stage in each node, A-TDOA architectures lead to better results than D-TDOA.

In summary, the A-TDOA system provides a higher accuracy than the D-TDOA method, but the latter presents a lower level of variation in the evaluation for sensor location. Although, A-TDOA architectures present more hardware complexity, they sport less energy consumption due to the reduction of the signal travel. On the basis of the information gathered, it can be concluded that the best method for multiple sensor locations is the A-TDOA system.

## 6. Discussion

The new asynchronous TDOA architectures have led to a major improvement as a consequence of the reduction of the complexity in sensor networks and the increasing accuracy of time measurements over the last few years. These methodologies have been experiencing a growing importance in LPS with particular application in robot indoor navigation and unmanned aerial vehicles (UAVs).

Amongst the asynchronous architectures, A-TDOA and D-TDOA have taken special relevance, but their novelty assumed that no previous research on the suitability of these systems had been accomplished before. This means that these architectures have not been studied in an actual common environment in order to determine a comparison among their system errors that would allow us to select the best architecture under different conditions. The error bounds must be calculated through the Cramér–Rao Lower Bound estimator all over the domain. In this context, CRLB allows to determine the minimum achievable error of a locating system with independence from the positioning algorithm used. With this parameter, the determination of the best asynchronous architecture could be done in a particular context. The extension of the usage of the LPS forces the design of an environment where CRLB must be derived in a 3D context for the first time.

This derivation includes a path-loss model propagation which depends on the distances between emitter and receiver of the positioning signal. This leads to heteroscedastic noise variances consideration that particularly fits with LPS.

The goal of this article has allowed for the development of a new methodology in order to select the best system in different contexts.

## 7. Conclusions

High accuracy requirements in modern applications lead to positioning systems where noise uncertainties must be minimized. New asynchronous positioning architectures have supposed a revolution where positioning errors have been considerably reduced. In this paper, a methodology to select the suitability of two asynchronous TDOA systems based on a CRLB evaluation under a 3D, real environment was accomplished for the first time to the best of our knowledge.

This analysis was performed based on a CRLB comparison where the uncertainties of time measurements originated by noise were distance dependent. This resulted in heteroscedasticity in the variance associated with sensor range estimation. This real model allowed us to determine the best TDOA asynchronous architecture with positioning algorithm independence.

The results showed that the A-TDOA system provided generally less uncertainty in the positioning, regardless of the node distributions. Nevertheless, the D-TDOA system achieved a better level of homogenization by reducing the RMSE standard deviation in the domain. On the basis of the information gathered, and taking into account the CRLB, it can be concluded that the best method for sensor location is the A-TDOA system.

These aspects are being treated in the current investigation, where the node distribution would be optimized for CRLB via genetic algorithms, attaining a RMSE minimization at all discretization points in future works.

## Figures and Tables

**Table 1:** Architecture parameters for Cramér-Rao Lower Bound (CRLB) study. Communication links amongst elements of Asynchronous Time Difference of Arrival (A-TDOA) and Difference-Time Difference of Arrival (D-TDOA) systems are restricted to these principal parameters. They were selected due to their utilization in similar tracking applications in the aerospace industry [26,27].

Parameter	Magnitude
Frequency of emission	1090 MHz
Bandwidth	100 MHz
Transmission power	400 W
Mean noise power	−94 dBm

**Table 2:** Node distributions in meters. Five random node distributions were defined in order to analyze the accuracy level of A-TDOA and D-TDOA architectures based on their CRLB system definition. CRLB evaluation does not require a classification of WS and CS nodes.

Distributions		*x*	*y*	*z*
D.1	Sensor 1	249	242	3
	Sensor 2	254	759	4
	Sensor 3	576	500	3
	Sensor 4	811	124	13
	Sensor 5	879	819	13
D.2	Sensor 1	72	156	3
	Sensor 2	141	854	13
	Sensor 3	496	484	3
	Sensor 4	810	891	3
	Sensor 5	876	133	13
D.3	Sensor 1	78	911	13
	Sensor 2	244	241	13
	Sensor 3	516	539	3
	Sensor 4	624	655	13
	Sensor 5	810	891	3
D.4	Sensor 1	191	880	10
	Sensor 2	435	527	3
	Sensor 3	482	198	9
	Sensor 4	758	254	3
	Sensor 5	782	788	7
D.5	Sensor 1	148	313	3
	Sensor 2	469	621	13
	Sensor 3	550	500	3
	Sensor 4	750	218	13
	Sensor 5	783	944	3

**Table 3:** RMSE distribution parameters for the five sensor distribution schemes in Table 2 are presented. These data were obtained based on the spatial discretization technique shown in Figure 3 and Figure 4.

RMSE (dB)		A-TDOA	D-TDOA
D.1	Mean	−0.5791	2.1446
	Min	−9.3067	−8.2291
	Max	21.4022	19.1978
	SD	5.5552	4.6881
D.2	Mean	−0.9528	2.3131
	Min	−8.6153	−7.4459
	Max	13.4399	19.3637
	SD	4.6718	4.1344
D.3	Mean	0.0682	2.2769
	Min	−8.9972	−8.0968
	Max	13.2448	11.9421
	SD	4.9828	3.6965
D.4	Mean	0.2111	2.6452
	Min	−9.1009	−7.4856
	Max	17.3846	18.3655
	SD	5.7176	4.5620
D.5	Mean	0.2524	2.2070
	Min	−9.8805	−8.1964
	Max	13.3707	11.8791
	SD	5.0180	3.8402
Mean of Means RMSE		−0.2000	2.3174
Mean Standard Deviations RMSE		5.1891	4.1842
